# The pediatrician’s role in the follow-up of children/adolescents with mental and/or neurodevelopmental disorders

**DOI:** 10.1016/j.jped.2025.101492

**Published:** 2025-12-19

**Authors:** Magda Lahorgue Nunes

**Affiliations:** Escola de Medicina da Pontifícia Universidade Católica do Rio Grande do Sul (PUC-RS), Instituto do Cérebro (InsCer), Porto Alegre, RS, Brazil

The topic of the 2026 supplement of Jornal de Pediatria, “The Pediatrician’s role in the follow-up of children/adolescents with mental and/or neurodevelopmental disorders,” was carefully chosen not only for its current characteristics but also for its relevance.

Mental and neurodevelopmental disorders are the subject of much exposure due to their widespread dissemination, not only in academic media but mainly in social media. Their high prevalence generates a great deal of interest in the general population, as reflected in doubts/questions brought to the pediatric office. However, there is a lot of misinformation on the subject, and often a simplistic approach without a scientific basis or even fake news that “goes viral” causes unnecessary alarm and fear.

The pediatrician plays a fundamental role in the follow-up of these children and in managing the network of support professionals who care for them. Additionally, it is the pediatrician, in childcare visits, who raises the early diagnostic suspicion and initiates the investigation.

Considering these aspects, we have selected current topics from the daily practice of pediatric clinics for this supplement and have assembled a carefully selected “team” of specialists from different areas with extensive experience in the subject.

We begin with Araújo’s article [1], which focuses on warning signs (red flags) for neurodevelopmental delays, followed by three articles that address different aspects of Autism Spectrum Disorder (ASD). Vasconcelos et al. [2]. review the state-of-the-art in ASD, including its etiological puzzle, clinical characteristics, pathophysiological mechanisms, differential diagnosis, and therapeutic management. Castro et al. [3]. critically analyze the factors that influence prevalence estimates, considering methodological, clinical, etiological, and sociocultural determinants that shape epidemiological data and diagnostic practices. Lin et al. [4]. review the behavioral approach strategies used in the treatment of ASD with a critical view of quantity × quality.

Following this, several topics are addressed, such as Attention Deficit/Hyperactivity Disorder, Irritability and Mood Disorders, Depression and Anxiety. Casella and Casella[5] present a narrative review on the pathophysiology, clinical aspects, differential diagnosis and therapeutic management of ADHD, highlighting the pediatrician’s central role in diagnostic suspicion and in the coordination of multidisciplinary care. De Oliveira et al. [6]. provide a comprehensive review of the “non-episodic irritability” complaint, intertwining diagnostic aspects and updated clinical recommendations. Linan et al. [7]. review the epidemiology, diagnostic criteria, and therapeutic options for the main anxiety and depressive disorders in childhood and adolescence.

There is ample evidence demonstrating a bidirectional relationship between sleep and mental/neurodevelopmental disorders. The impact of poor sleep quality permeates these disorders, and in the article by El Halal and Nunes [8], the presentation and management of sleep problems in affected children/adolescents is discussed.

Non-pharmacological therapeutic approaches are frequently described and indicated in the management of these disorders. Kostenko et al. [9]. analyze the current scientific literature on the main dietary and nutritional interventions proposed for children and adolescents with neuropsychiatric disorders and describe their efficacy and safety, differentiating evidence-based practices from common myths. Chaves et al. [10]. dissect the myths and truths, based on scientific evidence, of other recommended therapies/interventions, including transcranial stimulation, virtual reality, music therapy, and equine therapy.

Pfeifer [11] assesses the impact of urban violence, an increasingly present reality in our environment, on the mental health of children and adolescents.

Da Costa et al. [12]. discuss how neuropsychologists can assist in constructing a diagnosis of these disorders and show, in a didactic way, the most commonly used neuropsychological tests and how to interpret them.

We conclude the supplement with the article by Silva and Eisenstein [13], which addresses the transition to adolescence of a child with Neurodevelopmental Disorders. They review health care, the continuity of educational support, and the importance of individualized transition strategies, highlighting the need for a multidisciplinary approach, centered on the individual who is this neurodivergent adolescent.

We hope this supplement will serve as a guide for Pediatricians not only in diagnostic matters, but also in the management and follow-up of these children and adolescents.

Enjoy your reading!

*The Editor.*

## Data availability statement

Does not apply.
